# A surrogate BSL2-compliant infection model recapitulating key aspects of human Marburg virus disease

**DOI:** 10.1080/22221751.2024.2449083

**Published:** 2025-01-02

**Authors:** Wanying Yang, Wujie Zhou, Bo Liang, Xiaojun Hu, Shen Wang, Zhenshan Wang, Tiecheng Wang, Xianzhu Xia, Na Feng, Yongkun Zhao, Feihu Yan

**Affiliations:** a State Key Laboratory of Pathogenic Microorganisms, Key Laboratory of Jilin Province for Zoonosis Prevention and Control, Changchun Veterinary Research Institute, Chinese Academy of Agricultural Sciences, Changchun, People’s Republic of China; b International Joint Research Center of National Animal Immunology, College of Veterinary Medicine, Henan Agriculture University, Zhengzhou, People’s Republic of China; c College of Veterinary Medicine, Jilin Agricultural University, Changchun, People’s Republic of China

**Keywords:** Marburg virus, recombinant vesicular stomatitis virus, Syrian hamster, surrogate model, recurrence of classic symptoms, vaccine evaluation and drug screening

## Abstract

Marburg virus disease (MVD) is a severe infectious disease caused by the Marburg virus (MARV), posing a significant threat to humans. MARV needs to be operated under strict biosafety Level 4 (BSL-4) laboratory conditions. Therefore, accessible and practical animal models are urgently needed to advance prophylactic and therapeutic strategies for MARV. In this study, we constructed a recombinant vesicular stomatitis virus (VSV) expressing the Marburg virus glycoprotein (VSV-MARV/GP). Syrian hamsters infected with VSV-MARV/GP presented symptoms such as thrombocytopenia, lymphopenia, haemophilia, and multiorgan failure, developing a severe systemic disease akin to that observed in human MARV patients. Notably, the pathogenicity was found to be species-specific, age-related, sex-associated, and challenge route-dependent. Subsequently, the therapeutic efficacy of the MR191 monoclonal antibody was validated in this model. In summary, this alternative model is an effective tool for rapidly screening medical countermeasures against MARV GP in vivo under BSL-2 conditions.

## Introduction

Marburg virus (MARV), belonging to the family *Filoviridae* and genus *Marburgvirus*, is a highly pathogenic virus that causes severe illness, with a mortality rate of 23%–90% [1]. Among the various strains of MARV, Angola is significantly more pathogenic than the others. This finding has been primarily demonstrated in experiments with nonhuman primates (NHPs) [2–5]. Since 1967, when Marburg virus disease (MVD) was discovered, sporadic and episodic cases have been reported in various locations [6,7]. Notably, a severe outbreak occurred in 2004–2005, resulting in 252 infections, 227 of which were fatal, corresponding to a mortality rate of 90% [8]. The most recent MVD outbreak occurred in 2023 in Equatorial Guinea, generating significant global concern regarding MARV [9]. The transmission of MARV to humans primarily occurs through contact with infected animals. Furthermore, human-to-human transmission occurs through direct contact with infected individuals’ blood or other body fluids [2,10,11]. After a 2–21 days incubation period, symptoms of the disease manifest suddenly, including flu-like symptoms, fever, loss of appetite, vomiting, and weight loss, followed by maculopapular rash, hemorrhage, and ultimately shock, multiorgan failure, and death [12]. MARV is a non-segmented, negative-sense virus that contains a 19.1 kb RNA genome encoding seven genes, among which the glycoprotein gene (GP) is the only surface membrane protein responsible for mediating attachment to target cells and viral entry. Given this, the GP is a significant target for studying the pathogenesis of MVD and developing prophylactic vaccines and therapeutic antibodies against MARV.

Animal models capable of replicating the symptoms of MVD are invaluable tools for studying the pathogenesis and transmission of the disease, and enhancing preparedness for potential MVD outbreaks. MARV animal models are analogous to those used for Ebola virus (EBOV) and primarily include mice, guinea pigs, hamsters, ferrets, and NHPs [13]. Wild-type (WT) MARV is nonpathogenic in adult mice with intact immune systems but is lethal to suckling mice, severe combined immunodeficiency (SCID) mice, and transcription factor STAT1-knockout (*STAT1^-^*^/*-*^) mice [14–17]. This limitation hinders in vivo vaccine evaluation. Viral adaptation, a time-consuming process that may introduce additional mutations, is necessary for effective infection in guinea pigs and Syrian hamsters [18,19]. Ferrets do not develop disease when infected with MARV via various routes and doses [20,21]. NHPs, such as crab-eating and rhesus monkeys, more accurately replicate the disease processes and pathological changes observed in human infections. However, ethical concerns, high costs, and the limited availability of experimental facilities and reagents constrain the use of NHPs [22–26]. In contrast, surrogate models are able to simulate MVD symptoms and screen for medical countermeasures under BSL-2, which are more feasible and economical.

This study successfully rescued a recombinant vesicular stomatitis virus expressing the MARV glycoprotein, termed VSV-MARV/GP, and can serve as a surrogate virus for MARV infection in hamsters. Hamsters infected with VSV-MARV/GP exhibit critical characteristics, which are similar to MVD patients, with lethality closely associated with sex, age, and route of infection. In addition, the therapeutic efficacy of the MR191 monoclonal antibody was assessed in this model, demonstrating its utility. In conclusion, this study offers a valuable tool for screening anti-MARV GP vaccines and antibodies under BSL-2 conditions.

## Materials and methods

### Cells, viruses, and antibodies

Vero E6 cells (ATCC®CRL-1587^TM^) were stored at the Changchun Veterinary Institute and cultured in Dulbecco’s modified Eagle’s medium (DMEM; Gibco, Grand Island, NY, USA) supplemented with 10% fetal bovine serum (FBS; Gibco, Thermo Fisher Scientific, MA, USA) and 1% Pen-Strep in a 37°C, 5% CO_2_ incubator for culture. Recombinant VSV were generated by replacing the VSV glycoprotein with full-length GPs from MARV (GenBank NO. KR867677.1), termed VSV-MARV/GP, and were passaged in Vero E6 cells and stored at −80°C. WT VSV (GenBank NO. OR712768.1), murine anti-MARV antibody, and MR191 were stored at the Changchun Veterinary Institute.

### Construction and rescue of VSV-MARV/GP

**Figure 1.**
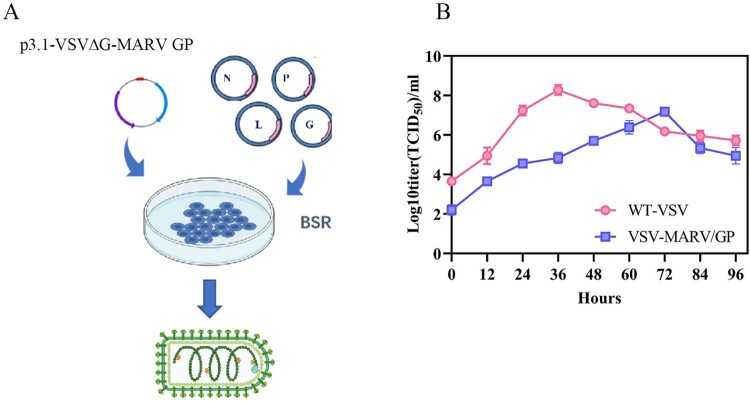
The generation and the One-step growth curves of the recombinant vesicular stomatitis virus VSV-MARV/GP. (**A**) Schematic diagram of the generation of the recombinant vesicular stomatitis virus VSV-MARV/GP. The recombinant full-length plasmid (p3.1-VSVΔG-MARV GP) and four helper plasmids (pcDNA3.1-VSV-N, pcDNA3.1-VSV-P, pcDNA3.1-VSV-L, and pcDNA3.1-VSV-G) were co-transfected into BSR/T7 cells to obtain a recombinant virus bearing the glycoprotein of MARV. (**B**) One-step growth curves of WT VSV and VSV-MARV/GP inoculated into Vero E6 cells at an MOI of 0.1. Viruses were collected at 12 h intervals to measure titers.

As we reported previously, a full-length VSV plasmid, named p3.1-VSV-eGFP, served as the tool [27]. The recombinant plasmid (named p3.1-VSVΔG-MARV GP) was generated by replacing the glycoprotein of VSV with the MARV full-length GP (NO. KR867677.1) and inserting an enhanced green fluorescent (eGFP) reporter gene between the N and P genes. Recombinant plasmids with four helper plasmids, pcDNA3.1-VSV-N, pcDNA3.1-VSV-P, pcDNA3.1-VSV-L, and pcDNA3.1-VSV-G, were cotransfected into BSR/T7 cells according to the calcium phosphate transfection kit instructions (Invitrogen, Waltham, MA, USA). Recombinant virus rescue was determined by cytopathological effects (CPEs) and expression of the eGFP reporter gene. The obtained recombinant viruses were passaged in Vero E6 cells (Figure 1(A)).

### In vitro growth kinetics of viruses

Vero E6 cells were cultured overnight in 24-well plates and infected separately with WT VSV or VSV-MARV/GP at a multiplicity of infection (MOI) of 0.1. The cell culture supernatants were collected at 12, 24, 48, 60, 72, 84, and 96 h post-infection (hpi), and viral titers were determined using the Reed-Muench method. A growth kinetics curve was then constructed, with time on the x-axis and viral titer on the y-axis (Figure 1(B)).

### BSL-2 containment animal experiments

Syrian hamsters, BALB/c mice, and Sprague‒Dawley (SD) rats were purchased from Beijing Vital River Laboratory Animal Technology Company Limited (Peking, CN). Hartley guinea pigs were purchased from Beijing Keyu Animal Breeding Center (Peking, CN). All animals were housed in a special pathogen-free facility with a 12:12-hour light‒dark cycle on sterile chow and sterilized water.

For common rodent infection experiments, sets of four (*n* = 5 per group) 5-week-old female SD rats, BALB/c mice, guinea pigs or Syrian hamsters were inoculated intraperitoneally with 10^7^ the 50% Tissue Culture Infectious Dose (TCID_50_) of VSV-MARV/GP, whereas control animals were injected intraperitoneally with the same volume of PBS.

To observe the post-infection characteristics of Syrian hamsters, 5-week-old female Syrian hamsters (*n* = 10 per group) were inoculated intraperitoneally with 10^7^ TCID_50_ of VSV-MARV/GP. Blood samples were collected at 36 hpi and analyzed for complete blood count and blood chemistry. To assess viral tissue tropism, the heart, liver, spleen, lung, kidney, stomach, intestine, and brain were also harvested and analyzed for pathology and viral distribution.

To determine the effect of sex on the mortality of Syrian hamsters infected with VSV-MARV/GP, 5-week-old male and female Syrian hamsters (*n* = 8 per group) were intraperitoneally inoculated with 10^7^ TCID_50_ VSV-MARV/GP. After 36 hpi, blood samples were collected for complete blood count and blood chemistry analysis. Three animals in each group were sacrificed, and liver, spleen, lung, and kidney samples were taken to assess virus distribution and pathology.

To investigate the influence of age on the lethality of Syrian hamsters infected with VSV-MARV/GP, 5-week-old, 3-month-old, and 1-year-old female Syrian hamsters (*n* = 8 per group) were subjected to intraperitoneal infection with 10^7^ TCID_50_ VSV-MARV/GP. After 36 hpi, blood samples were collected for complete blood counts and blood biochemical analyses, three animals in each group were sacrificed, and samples from the liver, spleen, lungs, and kidneys were collected to assess viral distribution and pathological changes.

To assess the role of the attack route on the lethality of Syrian hamsters infected with VSV-MARV/GP, four groups of 5-week-old Syrian hamsters were infected with VSV-MARV/GP via the intraperitoneal (i.p.), subcutaneous (s.c.), intramuscular (i.m.), or intranasal (i.n.) routes. At 36 hpi, blood samples were collected through the orbital venous plexus and subjected to complete blood count and blood chemistry analyses. Concurrently, visceral organs, including the liver, lungs, spleen, and kidneys were examined for the virus titers and pathology.

All animals were monitored daily for signs of illness, including weight, physical activity, and food and water intake changes.

### LD_50_ determination

The recombinant virus VSV-MARV/GP was diluted 10-fold to 10^4.5^–10^7.5^ TCID_50_/mL. Sixteen Syrian hamsters were randomly divided into four groups of four each, anesthetized with isoflurane, and then infected with different doses of VSV-MARV/GP via the i.p. route. Following infection, survival was monitored daily, body weight changes were recorded, and LD_50_ values were calculated based on group-specific mortality. Specifically, the LD_50_ was calculated using the modified Kärber method [28].

### Blood counts and blood biochemistry

To perform hematological tests in Syrian hamsters, blood was collected through the orbital venous plexus and placed into anticoagulant tubes containing EDTAK2. Complete blood counts and blood biochemistry analyses were performed with an automatic blood analyzer (BC-5000vet, Mindray, China), and the operation procedures were carried out according to the instrument's instructions.

### Quantification of viral loads by TCID_50_

The tissues were homogenized and removed from the cellular debris by centrifugation. The supernatant was serially diluted at a 10-fold ratio, and 100 μL was added to 96-well plates pre-lined with Vero E6 cells. The plates were incubated at 37°C for one hour and washed thrice with sterile PBS. One hundred microlitres of incomplete medium containing 2% FBS and 1% penicillin–streptomycin was added to each well of a 96-well plate. To calculate the median TCID_50_, the number of wells with a cytopathic effect (CPE) based on the Reed–Muench method was determined after incubation for 72 h [29].

### Histopathology and immunohistochemistry

Syrian hamsters were euthanized at the designated times, and tissues, including heart, liver, spleen, lungs, kidneys, stomach, intestines, and brain, were collected. The tissue samples were fixed in 10% neutral buffered formalin, cut, paraffin-embedded, sectioned on a 5-micron microtome, placed on slides, and stained with hematoxylin and eosin (H&E) for histopathological examination. A 10-minute incubation in a solution of 3% hydrogen peroxide in methanol was performed to prepare the paraffin-embedded tissues for immunohistochemical analysis. Specific anti-MARV GP immunoreactivity was then detected using a murine anti-MARV antibody as the primary antibody. Following incubation with the primary antibody, the sections were washed three times with PBS, and species-matched secondary antibodies were applied for two hours at 4°C. As previously described, histopathology and immunohistochemistry assays were performed [30]. Pathology scores according to the International Norms for the Terminology of Pathological Changes and Diagnostic Criteria in Rats and Mice (INHAND). Histochemical score (IRS) = SI (strength of positivity) × PP (percentage of positive cells). SI is classified into three levels, with no positive color at level 0, weakly positive color at level 1, moderately positive color at level 2, and strongly positive color at level 3; PP is classified into four levels, with 0%–5% at level 0, 6%–25% at level 1, 26%–50% at level 2, 51%–75% at level 3, and > 75% at level 4 [31]. The percentage of positive cells = number of positive cells/total number of cells [32].

### Evaluation of the MR191 antibody in the Syrian hamster model

Twenty-two female 5-week-old Syrian hamsters were randomized into two groups. All hamsters were challenged with 10^7^ TCID_50_ of VSV-MARV/GP in 1 mL of DMEM by intraperitoneal injection. At 6 hpi, Syrian hamsters in the treatment group were treated intraperitoneally with MR191 antibody (25 mg/kg). Similarly, all Syrian hamsters in the control group were given equal PBS. All animals were monitored daily for signs of disease, including changes in weight, physical activity, and food and water intake. At 36 hpi and 5 dpi, three Syrian hamsters were selected randomly from each group, and blood samples were collected through the orbital venous plexus to determine blood changes. Subsequently, euthanasia was performed, and the liver, spleen, lungs, and kidneys were collected for determination of the viral load, immunohistochemistry, and pathological changes.

### Statistical analysis

All the data were analyzed via GraphPad Prism v9.0 (GraphPad Software, Inc., San Diego, CA, USA) and are expressed as the mean standard error of the mean (SEM). One-way repeated measures analysis of variance (ANOVA) was used to compare changes in complete blood count and liver parameters. *p* <0.05 was considered a statistically significant difference. *, *P* < 0.05; **, *P* < 0.01; ***, *P* < 0.001; ****, *P* < 0.0001.

## Results

### VSV-MARV/GP is only lethally infectious in Syrian hamsters among small rodents

**Figure 2.**
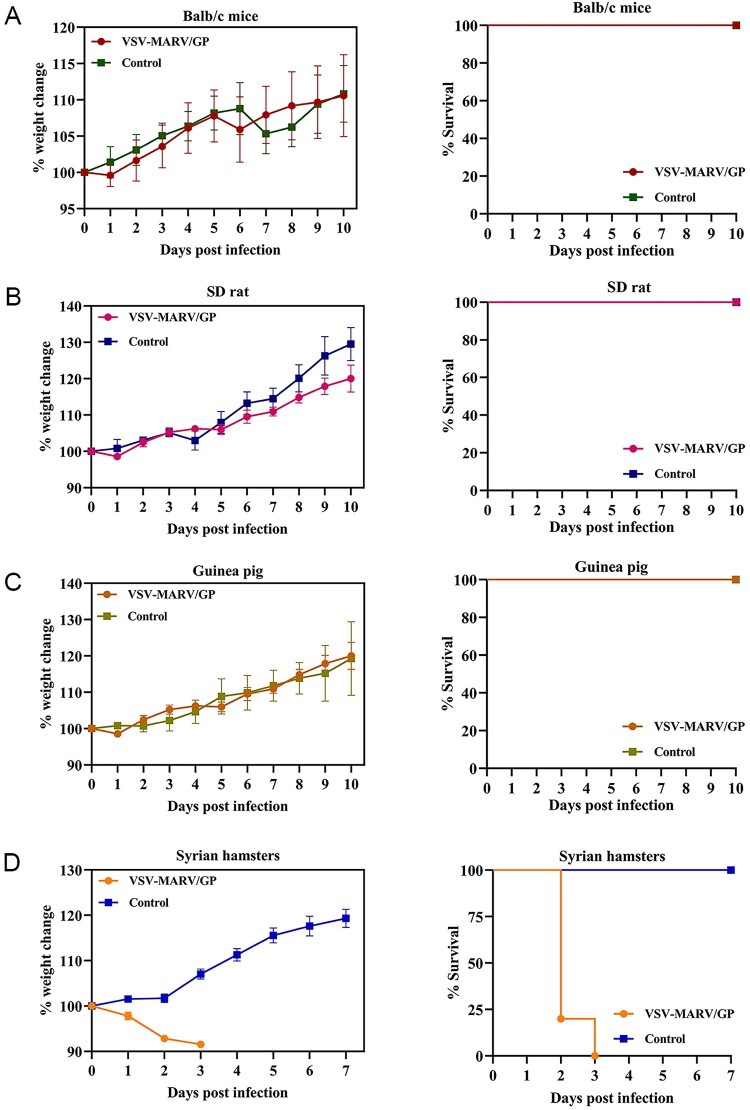
Changes in weight and percent survival of rodents infected with VSV-MARV/GP. BALB/c mice (**A**), Sprague‒Dawley (SD) rats (**B**), Hartley guinea pigs (**C**), and Syrian hamsters (**D**) were inoculated with VSV-MARV/GP via the intraperitoneal (i.p.) route. Weight changes and percent survival were monitored.

Initially, the sensitivities of SD rats, BALB/c mice, Hartley guinea pigs, and Syrian hamsters to VSV-MARV/GP were compared. The experimental groups of animals were treated with 10^7^ TCID_50_ of VSV-MARV/GP via i.p. injection, while the control groups were given the same volume of PBS; subsequently, each group was monitored daily for post-infection signs. At the end of the experiment, the SD rats, BALB/c mice, and Hartley guinea pigs survived and gained 10%–30% of their weight (Figure 2(A)–(C)). However, infected Syrian hamsters experienced approximately 10% weight loss, and all died from the disease within 2–3 dpi; however, the Syrian hamsters in the control group survived and were euthanized at 10 dpi as planned (Figure 2(D)). The Syrian hamster can be an alternative lethal model for infection with VSV-MARV/GP.

### Characterization of Syrian hamsters infected with VSV-MARV/GP

**Figure 3.**
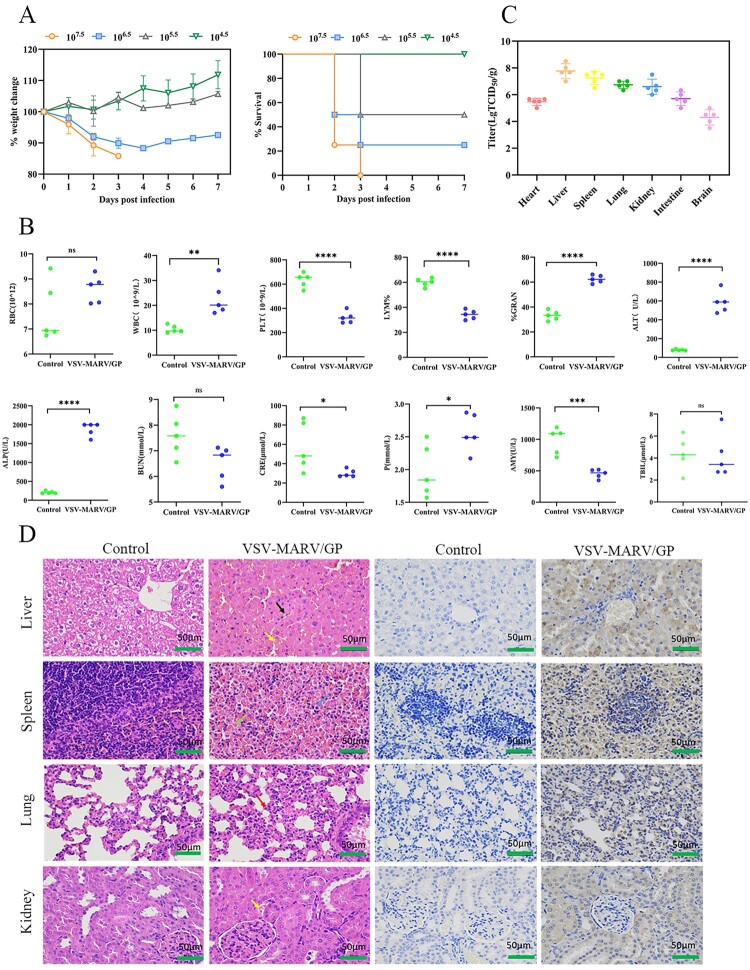
Characterization of VSV-MARV/GP infection in Syrian hamsters. (**A**) Weight change and survival of Syrian hamsters infected with different doses of VSV-MARV/GP. (**B**) Blood biochemistry and blood cell counts were analyzed at 36 hpi. (**C**) Viral loads, including those in the heart, liver, spleen, lungs, kidneys, stomach, intestines, and brain, were determined at 36 hpi. The data are presented as the means ± SEMs. Statistical analyses were performed via one-way ANOVA. *, *P* < 0.05; *, *P* < 0.01; **, *P* < 0.001; ***, *P* < 0.0001; ****. (**D**) Histopathological and immunohistochemistry assays of the liver, spleen, lung and kidney at 36 hpi. Scale bar = 50 μm. Hepatic lesions, including hepatocellular necrosis, nuclear fragmentation (black arrows) and hemorrhage (yellow arrows), were observed. Splenic lesions, including infiltrating macrophages (blue arrows) and hemorrhages (green arrows), were observed. The lung tissue showed diffuse mild thickening of the alveolar wall with inflammatory cell infiltration (red arrows). Kidney lesions were observed as hemorrhages (yellow arrows).

To further characterize the VSV-MARV/GP-infected Syrian hamsters, four groups of 5-week-old female Syrian hamsters were infected with different doses of VSV-MARV/GP (10^4.5^, 10^5.5^, 10^6.5^ TCID_50_, or 10^7.5^ TCID_50_). Overall, the mortality rate of Syrian hamsters increased with increasing infective dose, from nonlethal to 100% mortality. Among these, 10^7.5^ TCID_50_-infected hamsters had 100% mortality, and 10^5.5^ TCID_50_-infected hamsters had 50% mortality. Moreover, infected hamsters are commonly sequestered from their conspecifics and may manifest clinical signs such as diminished body weight, a recurved posture, lethargy, a rough coat, respiratory distress, and a diminished propensity for exploratory activities. The median lethal dose (LD_50_) of VSV-MARV/GP in Syrian hamsters was calculated to be approximately 5.6 × 10^5^ TCID_50_ via the modified Kärber method (Figure 3(A)).

Furthermore, Syrian hamsters (*n* = 5 per group) infected with VSV-MARV/GP were subjected to blood biochemical analyses at 36 hpi. Blood was collected from the orbital venous plexus and compared with the uninfected group. Specifically, markedly elevated concentrations of alkaline phosphatase (ALP) and aminotransferase (ALT) and decreased levels of amylase (AMY) were observed, indicating liver injury or pancreas-related disease. A significant increase in the granulocyte percentage (GRAN%), a moderate increase in the white blood cell (WBC) count, and a significant decrease in the platelet count (PLT) and lymphocyte percentage (LYM%) were also observed, suggesting severe viral infection and blood coagulation disorders. Parameters such as P, creatinine (CRE), blood urea nitrogen (BUN), total bilirubin (TBIL), and red blood cell count (RBC) showed minimal changes. These changes resemble the symptoms of MVD infection in humans [33] (Figure 3(B)).

To further investigate the replication of VSV-MARV/GP in Syrian hamster tissues, tissues, including heart, liver, spleen, lung, kidney, stomach, intestine, and brain, were harvested at 36 hpi to quantify the TCID_50_. The results revealed that VSV-MARV/GP was detectable in all the tissues tested, with the highest viral load in the liver (10^8.5^ TCID_50_/g) and the lowest in the brain (10^3.5^ TCID_50_/g) (Figure 3(C)).

In addition, pathological and histological analyses were performed to examine pathological changes in the liver, spleen, lungs, and kidneys at 36 hpi. In detail, necrosis and nuclear fragmentation were present in the hepatocytes, massive macrophage infiltration with hemorrhage in the red pulp of the spleen, mild thickening of the alveolar walls with diffuse lymphocyte infiltration was observed in many alveoli, and hemorrhage was observed in the kidneys. Antigen positivity was also detected in the liver, spleen, lungs, and kidneys, consistent with the viral load in the tissues and organs (Figure 3(D)).

Overall, 5-week-old Syrian hamsters infected with VSV-MARV/GP had a rapid onset and short period of disease, are predominantly hepatophilic, cause systemic infections, and develop a severe systemic.

### Sex-specific lethality in VSV-MARV/GP-infected Syrian hamsters

**Figure 4.**
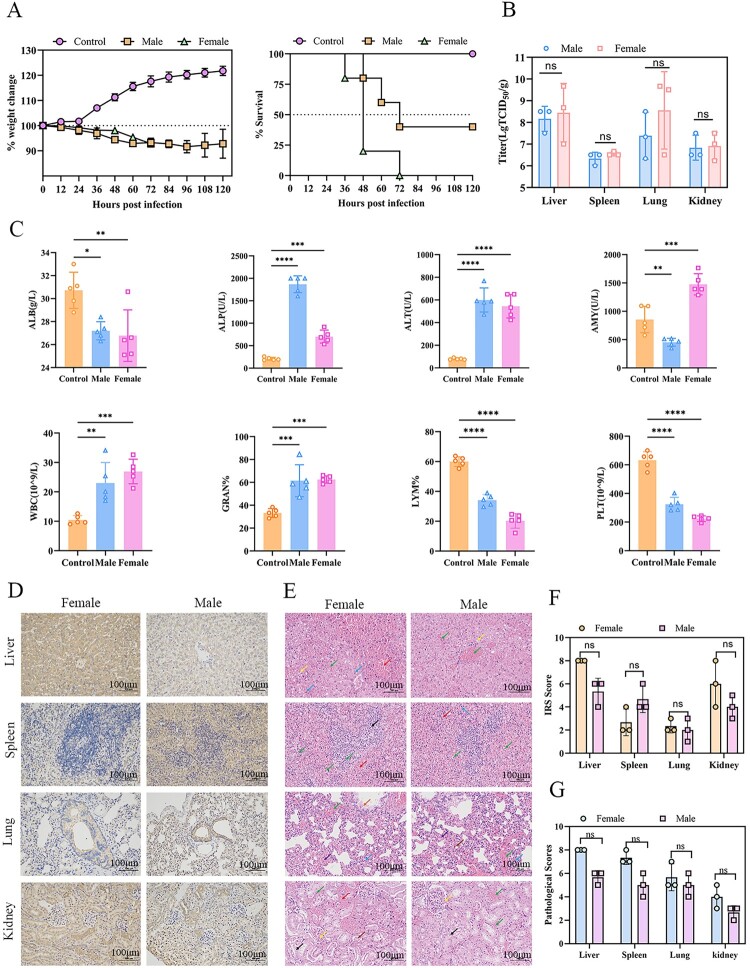
Comparison of sex-associated Syrian hamsters infected with VSV-MARV/GP.(**A**) Weight change and survival of the animals in the male and female groups. (**B**) Viral loads in the liver, spleen, lung and kidney at 1.5 dpi. (**C**) Hematological changes at 1.5 dpi. (**D**) Immunohistochemistry of the liver, spleen, lungs and kidneys at 1.5 dpi. Scale bar = 100 μm. (**E**) Histopathological changes in the liver, spleen, lungs and kidneys at 1.5 dpi. Scale bar = 100 μm. Pathological changes in the liver include hepatocellular steatosis (yellow arrows), venous and peripheral hepatic sinusoidal stasis (green arrows), hemorrhage (red arrows), and hepatocellular punctate necrosis (blue arrows). Splenic pathological changes include a low number, small size, and irregular shape of white marrow (black arrow), red marrow hemorrhage (red arrow), cellular necrosis (green arrow), and granulocyte infiltration (blue arrow). Pulmonary pathology includes granulocytic infiltration (blue arrow), congestion (purple arrow), bronchiole lumen eosinophilia (orange arrow), congestion (green arrow), and brown pigmentation (brown arrow). Renal pathological changes include glomerular capillary bruising (yellow arrows), hydropic degeneration of a small number of tubular epithelial cells (black arrows), tubular atrophy (brown arrows), vascular stasis (green arrows), and focal hemorrhage (red arrows). (**F**) IRS scores of the liver, spleen, lungs and kidneys at 1.5 dpi. (**G**) Pathological scores of the liver, spleen, lungs and kidneys at 1.5 dpi. The data are presented as the means ± SEMs. Statistical analyses were performed via one-way ANOVA. *, *P* < 0.05; *, *P* < 0.01; **, *P* < 0.001; ***, *P* < 0.0001; ****.

To understand the role of sex in lethality in VSV-MARV/GP-infected Syrian hamsters, three groups of 5-week-old Syrian hamsters (*n* = 10 per group), females, males and controls; females and males, were infected with 10^7^ TCID_50_ of VSV-MARV/GP, and controls were given the same volume of PBS. In the female group, all the Syrian hamsters succumbed to the disease within 36–72 hpi, with a weight loss of approximately 10%, whereas in the male group, the mortality rate was 60%, with some animals recovering from the infection. In the control group, all the Syrian hamsters survived and showed a sustained weight gain of 10%–20% (Figure 4(A)). At 36 hpi, blood was collected through the retro-orbital venous plexus for hematology; specifically, there were increased concentrations/activities of ALP, ALT, WBC, and GRAN% but decreased concentrations/activities of Albumin (ALB), PLT and LYM% (Figure 4(C)). Simultaneously, the liver, spleen, lungs, and kidneys were collected from each group of animals to determine tissue tropism. Overall, viral loads were more significant in the female group than in the male group, with the highest viral loads in the liver (Figure 4(B)). Heavy inflammatory cell infiltration, lymphocyte necrosis, and viral antigens were observed in the liver, spleen, lungs, and kidneys (Figure 4(D) and (E)). The female group had slightly higher pathological and histochemical scores than the male group did (Figure 4(F) and (G)). To increase the homogeneous lethality of VSV-MARV/GP-infected Syrian hamsters, female hamsters may be preferable.

### Age-related lethality in VSV-MARV/GP-infected Syrian hamsters

**Figure 5.**
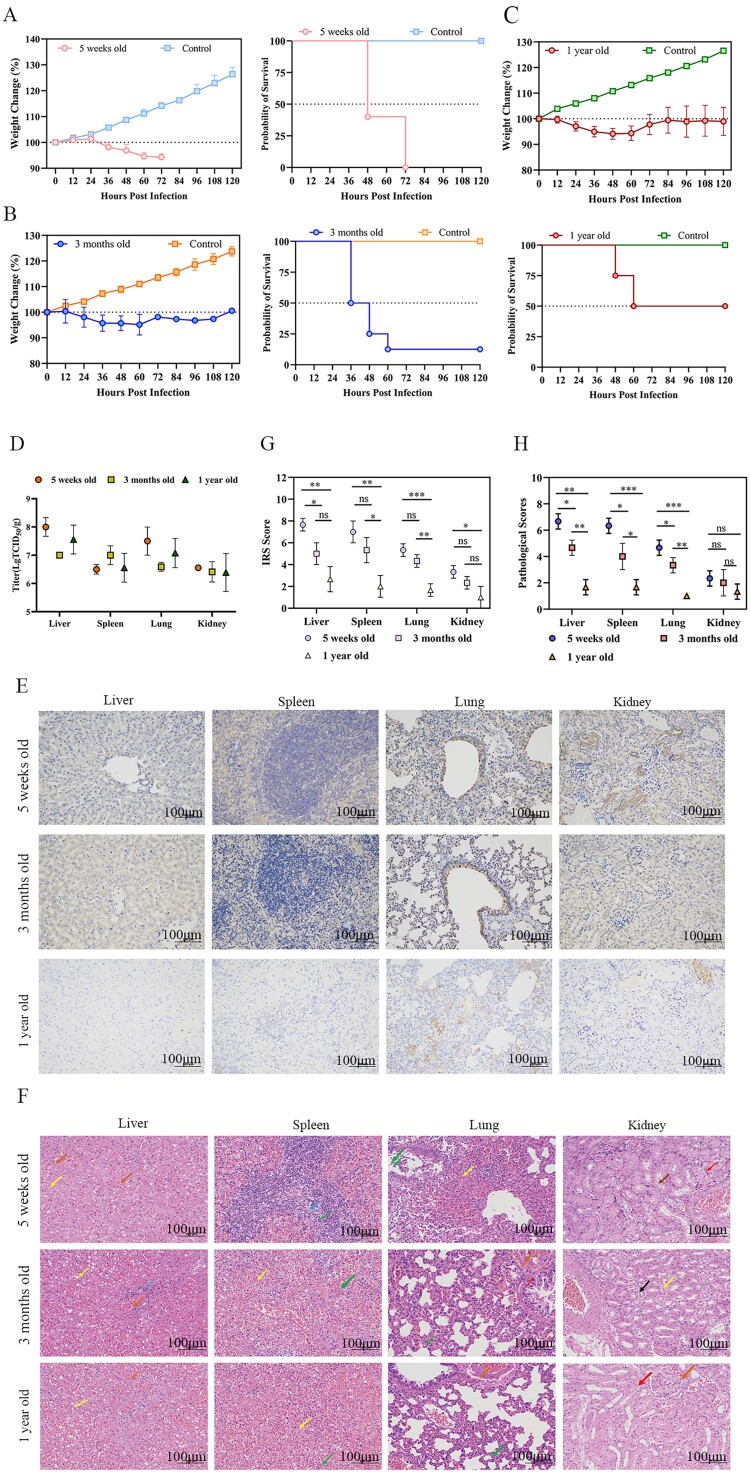
Comparison of the age distributions of Syrian hamsters infected with VSV-MARV/GP. Five-week-old (**A**), 3-month-old (**B**) and 1-year-old (**C**) Syrian hamsters were infected with VSV-MARV/GP via the i.p. route. Weight changes and percent survival were monitored. Viral loads were evaluated by TCID_50_ in the liver, spleen, lung and kidney at 1.5 dpi (**D**). Pathological changes (**E**) and immunohistochemistry (**F**) of the liver, spleen, lungs and kidneys 1.5 dpi. Hepatic pathology included hepatocellular steatosis (yellow arrows), venous and hepatic sinusoidal stasis (orange arrows) and focal infiltration of lymphocytes around the central vein (blue arrows). The splenic pathology revealed more sparsely arranged lymphocytes (blue arrows), granulocytic infiltration (green arrows) and hemorrhage (yellow arrows). Lung pathology included granulocytic infiltration (green arrows), bruising (orange arrows), and hydropic degeneration of fine bronchial epithelial cells (red arrows). Renal pathology includes hydropic degeneration of renal tubular epithelial cells (red arrows), intratubular eosinophilia (brown arrows), cytosolic consolidation of renal tubular epithelial cells (black arrows), detachment of renal tubular epithelial cells (yellow arrows), and bruising (orange arrows). Scale bar = 100 μm. IRS score (**G**) and pathological score (**H**). The data are presented as the means ± SEMs. Statistical analyses were performed via one-way ANOVA. *, *P* < 0.05; *, *P* < 0.01; **, *P* < 0.001; ***, *P* < 0.0001; ****.

To assess the impact of age on the lethality of VSV-MARV/GP-infected Syrian hamsters, we compared the outcomes in 5-week-old, 3-month-old, and 1-year-old female hamsters following i.p. infection. As a result, 5-week-old female Syrian hamsters exhibited 100% lethality following VSV-MARV/GP infection and died within 2–3 dpi, with a significant body weight loss of approximately 10% (Figure 5(A)). In contrast, the lethality observed in 3-month-old and 1-year-old hamsters infected with VSV-MARV/GP was variable, with rates of 87.5% and 50%, respectively. All hamsters experienced weight loss (approximately 10% of 3-month-olds and 7% of 1-year-olds, on average); however, some animals regained their original weight at 3 dpi (Figure 5(B)–(C)). At 36 hpi, the viral loads in the liver, spleen, lungs, and kidneys were assessed. Compared with the other two groups, the 5-week-old hamster group presented slightly greater overall viral loads, with the highest concentration observed in the liver (Figure 5(D)). Moreover, compared with the other two groups, the 5-week-old group presented more severe lesions in the liver, spleen, lungs, and kidneys, characterized by extensive inflammatory cell infiltration and viral antigens (Figure 5(E) and (F)). Additionally, this group had the highest pathological and histochemical scores (Figure 5(G) and (H)). For consistent lethality in VSV-MARV/GP-infected hamsters, selecting those that are 5 weeks old is preferable.

### Route-dependent lethality in VSV-MARV/GP-infected Syrian hamsters

**Figure 6.**
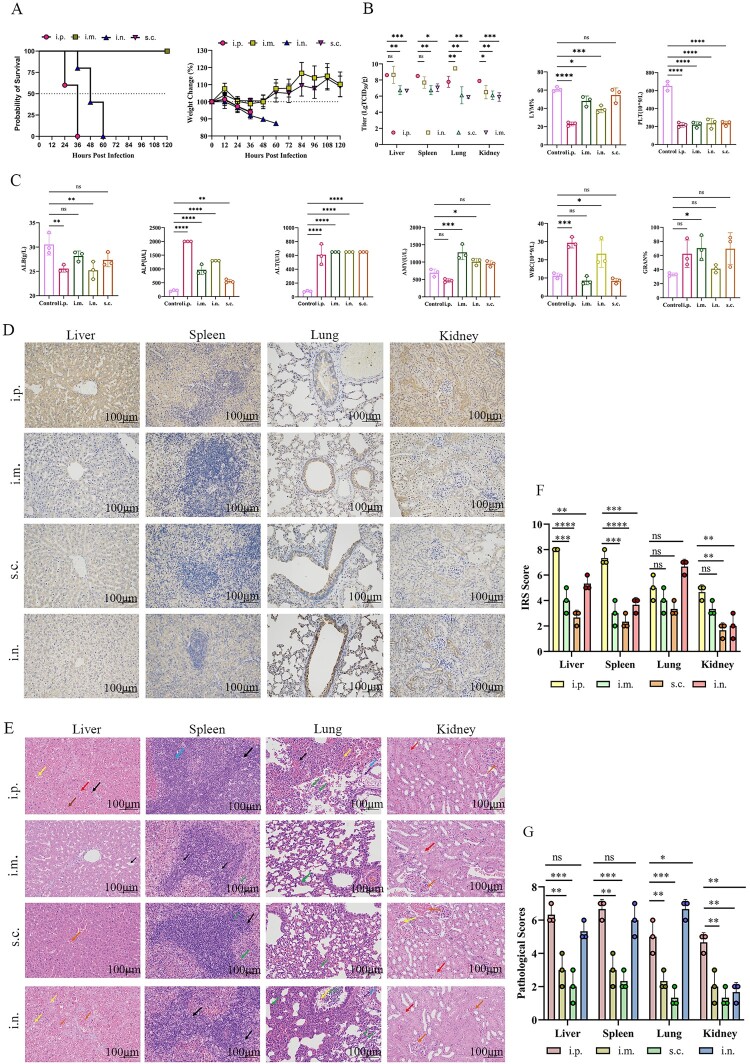
The effects of different challenge routes, including the intraperitoneal (i.p.), subcutaneous (s.c.), intramuscular (i.m.), and intranasal (i.n.) routes, on Syrian hamsters infected with VSV-MARV/GP were compared. (**A**) Weight change and survival of the animals in each group. (**B**) Viral load in the liver, spleen, lungs and kidneys at 1.5 dpi. (**C**) Hematological changes at 1.5 dpi. (**D**) Immunohistochemistry of the liver, spleen, lungs and kidneys at 1.5 dpi. Scale bar = 100 μm. (**E**) Histopathological changes in the liver, spleen, lung and kidney at 1.5 dpi. Scale bar = 100 μm. Hepatic pathological changes include dilated hepatic sinusoids (black arrows), hepatocellular steatosis (yellow arrows), hemorrhage (brown arrows), hepatocellular hydropic degeneration (red arrows), and venous and hepatic sinusoidal stasis (orange arrows). Splenic pathological changes include lymphocytic punctate necrosis (black arrows), granulocytic infiltration (green arrows), and white marrow adhesions (blue arrows). The pathological changes in the lungs included necrotic cell debris (black arrows), granulocytic infiltration (green arrows), lymphocytic infiltration (blue arrows), fine bronchial hemorrhage (yellow arrows) and perivascular edema (purple arrows). Renal pathological changes include hydropic degeneration of tubular epithelial cells (red arrows), glomerular capillary stasis (yellow arrows) and bruising (orange arrows). (**F**) Immunohistochemical scores of the liver, spleen, lungs and kidneys at 1.5 dpi. (**G**) Pathological scores of the liver, spleen, lungs and kidneys at 1.5 dpi.

To evaluate the effect of the infection route on the lethality of VSV-MARV/GP-infected Syrian hamsters, 5-week-old female hamsters were infected via the i.p., s.c., i.m., and i.n. routes. As a result, Syrian hamsters infected intraperitoneally died within 36 hpi, losing approximately 10% of their body weight. Those infected intranasally died within 60 hpi, with a body weight loss of approximately 15%. Hamsters infected via the s.c. and i.m. routes did not succumb and gradually recovered their body weight (Figure 6(A)). Blood biochemical analyses revealed increased concentrations/activities of ALP, ALT, WBC, and GRAN%, alongside decreased levels of ALB, PLT, and LYM%. The most pronounced changes were observed in the i.p. infection group, followed by the i.n. infection group (Figure 6(C)). In addition, viral loads were quantified in the liver, spleen, lung, and kidney of hamsters in each group (Figure 6(B)). Specifically, the viral loads in the liver were highest in the i.p. infection group, whereas those in the lungs were highest in the i.n. infection group. Moreover, the livers in the i.p. infection group and the lungs in the i.n. infection group presented the most severe lesions, with a significant presence of viral antigens compared with those in the other groups (Figure 6(D) and (E)). Pathological and immunohistochemical scores further corroborated these findings (Figure 6(F) and (G)). These results suggest that among the various routes of infection tested, both the i.p. and i.n. routes can lead to lethal infections. However, the i.n. route results in a slower progression of morbidity than the i.p. route does.

### MR191 protects Syrian hamsters against lethal infection with VSV-MARV/GP

**Figure 7.**
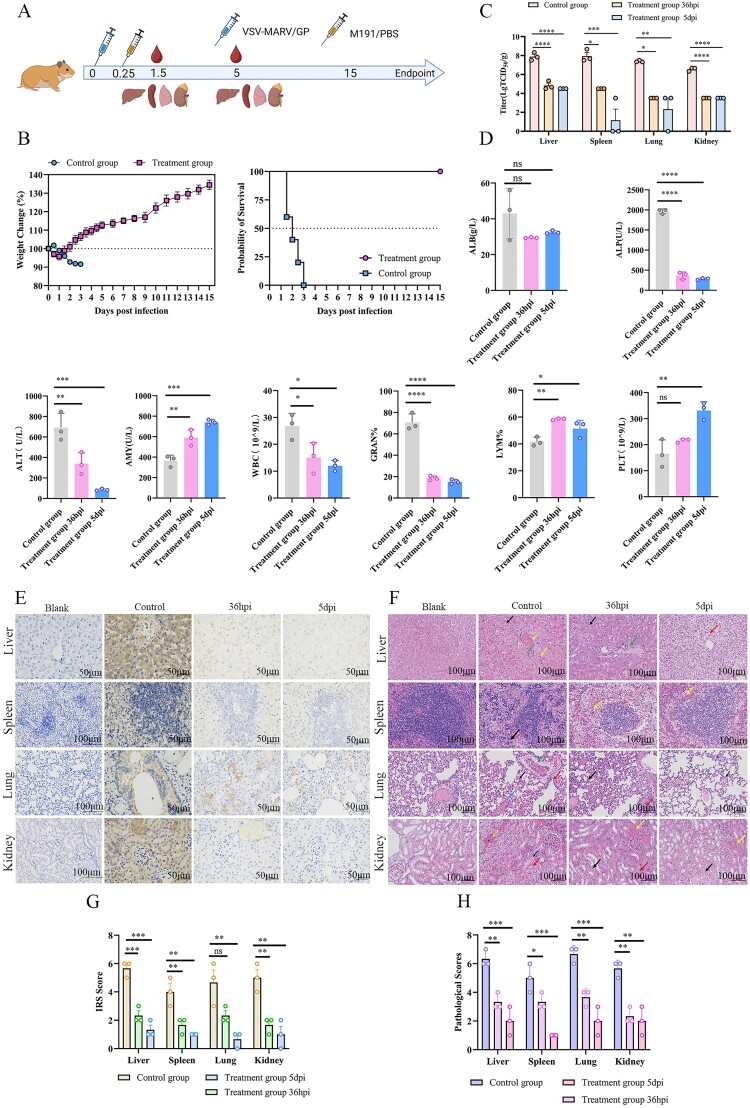
Assessment of the therapeutic efficacy of MR191 in a lethal Syrian hamster model. (**A**) Schematic diagram for assessing the therapeutic effect of MR191 in the Syrian hamster model. (**B**) Weight change and percent survival after VSV-MARV/GP infection. (**C**) Viral loads were evaluated by TCID_50_ in the liver, spleen, lung and kidney at 36 hpi and 5 dpi. (**D**) Hematological changes at 36 hpi and 5 dpi. (**E**) Immunohistochemistry of the liver, spleen, lungs and kidneys. Scale bar = 100 μm or 50 μm. (**F**) Pathological changes in the liver, spleen, lungs and kidneys. Scale bar = 100 μm. The hepatic lesions included hepatocellular ballooning (red arrows), hepatocellular edema (black arrows), bruising (yellow arrows), and lymphocytic infiltration (green arrows). Splenic lesions include cellular punctate necrosis (black arrows) and bruising (yellow arrows). Lung pathological changes include granulocyte infiltration (black arrows), edema of fine bronchial epithelial cells (red arrows), siltation of pulmonary veins and capillaries of alveolar walls (blue arrows), and perivascular edema (green arrows). Renal pathological changes include hydropic degeneration of tubular epithelial cells (red arrows), bruising (yellow arrows), and necrosis of tubular epithelial cells (black arrows). (**G**) IRS scores of the liver, spleen, lung and kidney. (**H**) Pathological scores of the liver, spleen, lung and kidney.

To evaluate the utility of VSV-MARV/GP-infected Syrian hamsters for in vivo screening of anti-MARV GP drugs, we investigated the therapeutic effects of passive immunization with MR191 in this model. MR191, a monoclonal antibody, has been shown to provide complete protection against MARV in nonhuman primates [34]. Furthermore, 5-week-old female hamsters were initially infected intraperitoneally with 10^7^ TCID_50_ of VSV-MARV/GP. Six hpi, the treatment group received an i.p. administration of MR191 (25 mg/kg), whereas the control group received an equivalent volume of PBS. Changes in body weight and survival were monitored for 15 days. At 36 hpi and 5 dpi, blood was collected via the retro-orbital venous plexus to assess hematological changes. Additionally, the liver, spleen, lungs, and kidneys were harvested for viral load, histopathological, and immunohistochemical analyses (Figure 7(A)). All hamsters in the control group died within 1.5–3 dpi and exhibited 10% weight loss. In contrast, all hamsters in the treatment group survived and began to regain their body weight at 1.5 dpi, ultimately exceeding 30% of their original body weight within 15 days of monitoring (Figure 7(B)). Compared with those in the control group, the levels of viral antigens in the liver, spleen, lungs, and kidneys progressively decreased with increasing duration of treatment (Figure 7(C)). As the duration of treatment increased, blood marker levels progressively approached normal values (Figure 7(D)). In the control group, the liver, spleen, lungs, and kidneys exhibited significant inflammatory cell infiltration, cellular necrosis, and viral antigens. In contrast, the treatment group demonstrated a substantial reduction in lesions, and viral antigens were progressively cleared with increasing duration of treatment (Figure 7(E) and (F)). The pathology and immunohistochemistry scores supported these findings (Figure 7(G) and (H)). These results suggest that the Syrian hamster model of VSV-MARV/GP infection serves as an effective tool for anti-MARV GP antibody evaluation.

## Discussion

MARV remains an ongoing threat to global public health. The sporadic occurrence of MVD outbreaks, coupled with their high degree of transmission and extreme lethality, has resulted in a limited understanding of the immune response and therapeutic interventions for MVD. No treatment or vaccine has been licensed to treat or prevent MVD. However, some promising countermeasures have been developed [35]. The absence of optimal animal models and the necessity for high-containment laboratories have impeded the study of MARV pathogenesis and the development of medical countermeasures. Therefore, developing suitable animal models for alternative MARV that can be utilized under BSL-2 conditions and accurately replicate the symptoms of human infection will accelerate technological advances and breakthroughs in the study of MVD.

As a well-characterized model virus, VSV has been extensively utilized in the development of viral vector vaccines, oncolytic viruses, and pseudo-virus packaging systems [36]. VSV is a non-segmented, negative-sense RNA virus that can be manipulated via reverse genetics techniques. Different viral tropisms are often determined by the interaction between viral envelope proteins and their host receptors. VSV exhibits robust pantropic infectivity mediated by its envelope protein, VSV G [37]. The most commonly used design strategy, VSVΔG, involves replacing the VSV G protein with an exogenous glycoprotein, which alters the cellular and tissue tropism of the recombinant virus, depending on the specific exogenous glycoprotein utilized [38,39]. VSV vectors have been applied for EBOV. VSV-EBOV can be assessed as a vaccine candidate in nonhuman primate models [36,40]. This study provides a theoretical basis for the rational application of recombinant VSV-based alternative viruses in MARV.

Previous studies have demonstrated that NHPs are susceptible to MARV and effectively replicate the symptoms of human infection, thereby serving as the gold standard models [30,41–45]. However, these animal models must be utilized in highly controlled laboratory environments under BSL-4. Compared with NHPs, small animal models are more convenient, cost effective, and readily accessible. Regrettably, conventional small animal models, including mice, hamsters, guinea pigs, and ferrets, exhibit either insensitivity or reduced sensitivity to MARV [18,20,46]. To overcome this challenge, lethal MARV infection has been facilitated through the use of virus-adapted or immunodeficient animal models [14,16,19,47,48]. Compared with the original virus, viral adaptation can introduce additional mutations during transmission, potentially altering pathogenicity [49]. Furthermore, immunodeficient animals are susceptible to fatal infections and have been utilized to evaluate virus-like particle vaccines; however, they are unsuitable for live vaccine assessment due to their compromised immune systems [14,15]. Similarly, the resistance of rodents to MARV presents a challenge for the use of a surrogate virus, as rodents may also resist it. Therefore, this study examined rodent infections caused by surrogate viruses, focusing on species specificity, age relevance, sex correlation, and determinants of the infectious pathway. Among all the animals tested, including BALB/c mice, SD rats, guinea pigs, and Syrian hamsters, 5-week-old female Syrian hamsters were lethally infected with VSV-MARV/GP through the i.p. and i.n. routes. Specifically, Syrian hamsters infected with VSV-MARV/GP presented key symptoms, which are similar to MVD patients, including weight loss, depression, respiratory distress, and loss of exploratory behaviour; uniform mortality; multiorgan failure; and abnormal blood parameters, such as low WBC and PLT counts, elevated ALP and ALT levels, and high viral loads, suggesting systemic replication and hepatophilic effects of the virus, all of which have been observed in MVD patients and NHPs [23,44,50,51]. Previous studies have shown that Syrian hamsters infected with hamster-adapted MARV (HA-MARV) display almost all of the clinical features of MVD observed in humans and nonhuman primates, including coagulation abnormalities, hemorrhagic manifestations, petechial rash, and a severely dysregulated immune response. Thrombocytopenia and elevated viral loads in vivo have also been observed, with histopathological changes, including inflammatory cell infiltration, cellular necrosis, apoptosis, and severe hepatic damage [19,43,52,53]. However, in our study, Syrian hamsters were infected with VSV-MARV/GP instead of HA-MARV, which fully replicated the aforementioned symptoms and could be handled under BSL-2 conditions for the rapid preclinical evaluation of anti-MARV GP antibodies and vaccines. Pseudo-viruses are crucial for developing animal models. In previous studies, pseudo-viruses were constructed to establish bioluminescent imaging mouse models using HIV as a vector with an incorporated reporter gene [54,55]. These models facilitate real-time observation and analysis of drug inhibitors’ effects and permit safe, effective in vivo assessment of drug activity without sacrificing the host [56]. However, the sensitivity of bioluminescence can be influenced by skin and tissue pigmentation, as well as organ depth [57–59]. Conversely, VSV-MARV/GP-infected hamsters present a lethal model, facilitating enhanced evaluation of therapeutic countermeasures’ effectiveness.

In addition, the model confirms the in vivo therapeutic efficacy of the MR191 antibody, which was previously validated in NHPs [60]. The MR191 antibody provides 100% protection to Syrian hamsters against lethal challenge with surrogate viruses. This was evidenced by a reduction in the viral load and pathological damage to the tissue. These results suggest that surrogate models could serve as a cost-effective approach for the initial and rapid screening of medical countermeasures against MARV.

In conclusion, we have developed a lethal model of the MARV surrogate virus with well-defined prerequisites and thorough validation; however, the underlying mechanisms contributing to morbidity require further investigation. For example, unlike our previously established model of lethality in Syrian hamsters intraperitoneally infected with VSV-EBOV/GP, both i.p. and i.n. administration resulted in the mortality of these hamsters; however, further elucidation of the underlying mechanisms is needed [61]. Previous studies have demonstrated that the pathogenicity of VSV-MARV/GP differs from that of VSV-EBOV/GP in ferrets, indicating that the inability of MARV to cause pathogenicity in ferrets is not solely linked to GP [62]. Therefore, we speculate that the receptor for MARV, which is distinct from that of Ebola virus (EBOV) -a related filovirus- comprises more than just Niemann-Pick C1 (NPC1) [63]. The accelerated disease progression and onset in hamsters infected via the i.p. route, compared to the i.n. route, can likely be attributed to the hepatophilic nature of MARV. This is facilitated by the prompt and efficient exposure of hepatocytes via the intraperitoneal route combined with the rapid replication capacity of VSV, ultimately resulting in multiple organ failure and a fatal outcome. We observed age-related lethality of VSV-MARV/GP in Syrian hamsters. Notably, 5-week-old female Syrian hamsters presented the greatest degree of lethality following infection with VSV-MARV/GP. Syrian hamster suckling mice have been reported to be susceptible to WT MARV infection, exhibiting neuropathological changes in the brain, including consistent observations of swelling, vasodilation, and hemorrhage [64]. This may be due to incomplete immunity in 5-week-old hamsters, which makes them less capable of combating the virus than adult Syrian hamsters are, resulting in more uniform lethality. We observed that lethality was more consistent in female hamsters than in male hamsters, potentially due to sex-based differences in the immune system; however, further validation is needed [65].

## Conclusions

In summary, this study develops a cost-effective and safe surrogate model for MVD that is consistently lethal and illustrates several key aspects of human MVD. This alternative virus, along with the corresponding hamster MVD model, plays a crucial role in the evaluation of MARV drugs and medical countermeasures under BSL-2 conditions. This makes preclinical assessments more convenient, rapid, and cost-effective. This hamster model represents a powerful tool for further dissecting MARV pathogenesis and accelerating the development of effective medical countermeasures against human MVD. However, the pathogenesis of VSV-MARV/GP infection in hamsters requires further investigation.

## References

[1] Abir MH, Rahman T, Das A, et al. Pathogenicity and virulence of Marburg virus. Virulence. 2022;13(1):609–633. doi:10.1080/21505594.2022.2054760 35363588 PMC8986239

[2] Brauburger K, Hume AJ, Mühlberger E, et al. Forty-five years of Marburg virus research. Viruses. 2012;4(10):1878–1927. doi:10.3390/v4101878 23202446 PMC3497034

[3] Geisbert TW, Daddario-DiCaprio KM, Geisbert JB, et al. Marburg virus Angola infection of rhesus macaques: pathogenesis and treatment with recombinant nematode anticoagulant protein c2. J Infect Dis. 2007;196(Suppl 2):S372–S381. doi:10.1086/520608 17940973 PMC7110112

[4] Daddario-DiCaprio KM, Geisbert TW, Geisbert JB, et al. Cross-protection against Marburg virus strains by using a live, attenuated recombinant vaccine. J Virol. 2006;80(19):9659–9666. doi:10.1128/JVI.00959-06 16973570 PMC1617222

[5] Alves DA, Glynn AR, Steele KE, et al. Aerosol exposure to the Angola strain of marburg virus causes lethal viral hemorrhagic fever in cynomolgus macaques. Vet Pathol. 2010;47(5):831–851. doi:10.1177/0300985810378597 20807825

[6] Hennessen W. A hemorrhagic disease transmitted from monkeys to man. Natl Cancer Inst Monogr. 1968;29:161–171. 5752977

[7] Cuomo-Dannenburg G, McCain K, McCabe R, et al. Marburg virus disease outbreaks, mathematical models, and disease parameters: a systematic review. Lancet Infect Dis. 2024;24(5):e307–e317. doi:10.1016/S1473-3099(23)00515-7 38040006 PMC7615873

[8] Towner JS, Khristova ML, Sealy TK, et al. Marburgvirus genomics and association with a large hemorrhagic fever outbreak in Angola. J Virol. 2006;80(13):6497–6516. doi:10.1128/JVI.00069-06 16775337 PMC1488971

[9] Samarasekera U. Marburg virus outbreak in Equatorial Guinea. Lancet Infect Dis. 2023;23(5):534. doi:10.1016/S1473-3099(23)00221-9 37086728

[10] Bausch DG, Borchert M, Grein T, et al. Risk factors for Marburg hemorrhagic fever, democratic republic of the Congo. Emerg Infect Dis. 2003;9(12):1531–1537. doi:10.3201/eid0912.030355 14720391 PMC3034318

[11] Chakraborty S, Chandran D, Mohapatra RK, et al. Sexual transmission of recently re-emerged deadly Marburg virus (MARV) needs explorative studies and due attention for its prevention and feasible spread - correspondence. Int J Surg (London, England). 2022;106:106884. doi:10.1016/j.ijsu.2022.106884 36075554

[12] Rougeron V, Feldmann H, Grard G, et al. Ebola and Marburg haemorrhagic fever. J Clin Virol. 2015;64:111–119. doi:10.1016/j.jcv.2015.01.014 25660265 PMC11080958

[13] Siragam V, Wong G, Qiu XG. Animal models for filovirus infections. Zool Res. 2018;39(1):15–24. doi:10.24272/j.issn.2095-8137.2017.053 29511141 PMC5869237

[14] Bray M. The role of the type I interferon response in the resistance of mice to filovirus infection. J Gen Virol. 2001;82(Pt 6):1365–1373. doi:10.1099/0022-1317-82-6-1365 11369881

[15] Raymond J, Bradfute S, Bray M. Filovirus infection of STAT-1 knockout mice. J Infect Dis. 2011;204(suppl_3):S986–S990. doi:10.1093/infdis/jir335 21987780

[16] Warfield KL, Alves DA, Bradfute SB, et al. Development of a model for marburgvirus based on severe-combined immunodeficiency mice. Virol J. 2007;4(1):108. doi:10.1186/1743-422X-4-108 17961252 PMC2164958

[17] Qiu X, Wong G, Audet J, et al. Establishment and characterization of a lethal mouse model for the Angola strain of Marburg virus. J Virol. 2014;88(21):12703–12714. doi:10.1128/JVI.01643-14 25142608 PMC4248893

[18] Banadyga L, Dolan MA, Ebihara H. Rodent-Adapted filoviruses and the molecular basis of pathogenesis. J Mol Biol. 2016;428(17):3449–3466. doi:10.1016/j.jmb.2016.05.008 27189922 PMC5010511

[19] Marzi A, Banadyga L, Haddock E, et al. A hamster model for Marburg virus infection accurately recapitulates Marburg hemorrhagic fever. Sci Rep. 2016;6:39214. doi:10.1038/srep39214 27976688 PMC5157018

[20] Cross RW, Mire CE, Agans KN, et al. Marburg and Ravn viruses fail to cause disease in the domestic ferret (mustela putorius furo). J Infect Dis. 2018;218(suppl_5):S448–s452. 29955887 10.1093/infdis/jiy268 PMC6249574

[21] Wong G, Zhang Z, He S, et al. Marburg and Ravn virus infections Do Not cause observable disease in ferrets. J Infect Dis. 2018;218(suppl_5):S471–s474. doi:10.1093/infdis/jiy245 29889278 PMC6249572

[22] Geisbert TW, Strong JE, Feldmann H. Considerations in the Use of nonhuman primate models of ebola virus and Marburg virus infection. J Infect Dis. 2015;212(Suppl 2):S91–S97. doi:10.1093/infdis/jiv284 26063223 PMC4564553

[23] Fernando L, Qiu X, Melito PL, et al. Immune response to Marburg virus Angola infection in nonhuman primates. J Infect Dis. 2015;212(Suppl 2):S234–S241. doi:10.1093/infdis/jiv095 25957966

[24] Finch CL, King TH, Alfson KJ, et al. Single-Shot ChAd3-MARV vaccine in modified formulation buffer shows 100% protection of NHPs. Vaccines (Basel). 2022;10(11):1935. doi:10.3390/vaccines10111935 36423030 PMC9694189

[25] Hunegnaw R, Honko AN, Wang L, et al. A single-shot ChAd3-MARV vaccine confers rapid and durable protection against Marburg virus in nonhuman primates. Sci Transl Med. 2022;14(675):eabq6364. doi:10.1126/scitranslmed.abq6364 36516269

[26] Marzi A, Jankeel A, Menicucci AR, et al. Single dose of a VSV-based vaccine rapidly protects macaques from Marburg virus disease. Front Immunol. 2021;12:774026. doi:10.3389/fimmu.2021.774026 34777392 PMC8578864

[27] Wang S, Zhang C, Liang B, et al. Characterization of immune response diversity in rodents vaccinated with a vesicular stomatitis virus vectored COVID-19 vaccine. Viruses. 2022;14(6):1127. doi:10.3390/v14061127. 35746599 PMC9227808

[28] Wan D, Zhou X, Xie C, et al. Toxicological evaluation of ferrous N-carbamylglycinate chelate: acute, Sub-acute toxicity and mutagenicity. Regul Toxicol Pharmacol. 2015;73(2):644–651. doi:10.1016/j.yrtph.2015.09.013 26364753

[29] Han Q, Wang S, Wang Z, et al. Nanobodies with cross-neutralizing activity provide prominent therapeutic efficacy in mild and severe COVID-19 rodent models. Virol Sin. 2023;38(5):787–800. doi:10.1016/j.virs.2023.07.003 37423308 PMC10590698

[30] Hensley LE, Alves DA, Geisbert JB, et al. Pathogenesis of Marburg hemorrhagic fever in cynomolgus macaques. J Infect Dis. 2011;204(suppl_3):S1021–S1031. doi:10.1093/infdis/jir339 21987738

[31] Xie P, Zhang M, He S, et al. The covalent modifier Nedd8 is critical for the activation of Smurf1 ubiquitin ligase in tumorigenesis. Nat Commun. 2014;5(1):3733. doi:10.1038/ncomms4733 24821572

[32] Benonisson H, Altıntaş I, Sluijter M, et al. CD3-Bispecific antibody therapy turns solid tumors into inflammatory sites but does Not install protective memory. Mol Cancer Ther. 2019;18(2):312–322. doi:10.1158/1535-7163.MCT-18-0679 30381448

[33] Shifflett K, Marzi A. Marburg virus pathogenesis - differences and similarities in humans and animal models. Virol J. 2019;16(1):165. doi:10.1186/s12985-019-1272-z 31888676 PMC6937685

[34] Rghei AD, Cao W, He S, et al. AAV-Vectored Expression of Marburg virus-neutralizing antibody MR191 provides complete protection from challenge in a Guinea Pig model. J Infect Dis. 2023;228(Suppl 7):S682–s690. doi:10.1093/infdis/jiad345 37638865 PMC10651196

[35] Bradfute SB. The discovery and development of novel treatment strategies for filoviruses. Expert Opin Drug Discovery. 2022;17(2):139–149. doi:10.1080/17460441.2022.2013800 34962451

[36] Wang S, Liang B, Wang W, et al. Viral vectored vaccines: design, development, preventive and therapeutic applications in human diseases. Signal Transduct Target Ther. 2023;8(1):149. doi:10.1038/s41392-023-01408-5 37029123 PMC10081433

[37] Tober R, Banki Z, Egerer L, et al. VSV-GP: a potent viral vaccine vector that boosts the immune response upon repeated applications. J Virol. 2014;88(9):4897–4907. doi:10.1128/JVI.03276-13 24554655 PMC3993835

[38] Poetsch JH, Dahlke C, Zinser ME, et al. Detectable vesicular stomatitis virus (VSV)-specific humoral and cellular immune responses following VSV-ebola virus vaccination in humans. J Infect Dis. 2019;219(4):556–561. doi:10.1093/infdis/jiy565 30452666 PMC6350948

[39] Ke Y, Zhang E, Guo J, et al. Immunogenicity of mucosal COVID-19 vaccine candidates based on the highly attenuated vesicular stomatitis virus vector (VSV(MT)) in golden Syrian hamster. Acta Pharmaceutica Sinica B. 2023;13(12):4856–4874. doi:10.1016/j.apsb.2023.08.023 38045049 PMC10692390

[40] Marzi A, Reynolds P, Mercado-Hernandez R, et al. Single low-dose VSV-EBOV vaccination protects cynomolgus macaques from lethal ebola challenge. EBioMedicine. 2019;49:223–231. doi:10.1016/j.ebiom.2019.09.055 31631035 PMC6945200

[41] Dye JM, Herbert AS, Kuehne AI, et al. Postexposure antibody prophylaxis protects nonhuman primates from filovirus disease. Proc Natl Acad Sci U S A. 2012;109(13):5034–5039. 22411795 10.1073/pnas.1200409109 PMC3323977

[42] Johnston SC, Lin KL, Twenhafel NA, et al. Dose response of MARV/Angola infection in Cynomolgus Macaques following IM or aerosol exposure. PLoS One. 2015;10(9):e0138843. 26413900 10.1371/journal.pone.0138843 PMC4586374

[43] Glaze ER, Roy MJ, Dalrymple LW, et al. A comparison of the pathogenesis of Marburg virus disease in humans and nonhuman primates and evaluation of the suitability of these animal models for predicting clinical efficacy under the ‘animal rule’. Comp Med. 2015;65(3):241–259. 26141449 PMC4485633

[44] Lin KL, Twenhafel NA, Connor JH, et al. Temporal characterization of Marburg virus Angola infection following Aerosol challenge in Rhesus Macaques. J Virol. 2015;89(19):9875–9885. doi:10.1128/JVI.01147-15 26202230 PMC4577920

[45] Wang S, Li W, Wang Z, et al. Emerging and reemerging infectious diseases: global trends and new strategies for their prevention and control. Signal Transduct Target Ther. 2024;9(1):223. doi:10.1038/s41392-024-01917-x 39256346 PMC11412324

[46] Cross RW, Fenton KA, Geisbert JB, et al. Comparison of the pathogenesis of the Angola and Ravn strains of Marburg virus in the outbred Guinea Pig model. J Infect Dis. 2015;212(Suppl 2):S258–S270. doi:10.1093/infdis/jiv182 26092858 PMC4564542

[47] Wei H, Audet J, Wong G, et al. Deep-sequencing of Marburg virus genome during sequential mouse passaging and cell-culture adaptation reveals extensive changes over time. Sci Rep. 2017;7(1):3390. doi:10.1038/s41598-017-03318-3 28611428 PMC5469859

[48] Atkins C, Miao J, Kalveram B, et al. Natural history and pathogenesis of wild-type Marburg virus infection in STAT2 knockout hamsters. J Infect Dis. 2018;218(suppl_5):S438–s447. 30192975 10.1093/infdis/jiy457 PMC6249581

[49] Lofts LL, Ibrahim MS, Negley DL, et al. Genomic differences between Guinea pig lethal and nonlethal Marburg virus variants. J Infect Dis. 2007;196(s2):S305–S312. doi:10.1086/520585 17940965

[50] Nicholas VV, Rosenke R, Feldmann F, et al. Distinct biological phenotypes of Marburg and Ravn virus infection in macaques. J Infect Dis. 2018;218(suppl_5):S458–s465. doi:10.1093/infdis/jiy456 30215737 PMC6249580

[51] Slenczka WG. The Marburg virus outbreak of 1967 and subsequent episodes. Curr Top Microbiol Immunol. 1999;235:49–75. 9893378 10.1007/978-3-642-59949-1_4

[52] Kortepeter MG, Bausch DG, Bray M. Basic clinical and laboratory features of filoviral hemorrhagic fever. J Infect Dis. 2011;204(suppl_3):S810–S816. doi:10.1093/infdis/jir299 21987756

[53] Mehedi M, Groseth A, Feldmann H, et al. Clinical aspects of Marburg hemorrhagic fever. Future Virol. 2011;6(9):1091–1106. doi:10.2217/fvl.11.79 22046196 PMC3201746

[54] Zhang L, Li Q, Liu Q, et al. A bioluminescent imaging mouse model for Marburg virus based on a pseudovirus system. Hum Vaccin Immunother. 2017;13(8):1811–1817. doi:10.1080/21645515.2017.1325050 28481728 PMC5557217

[55] Lei S, Huang W, Wang Y, et al. In vivo bioluminescent imaging of Marburg virus in a rodent model. Methods Mol Biol (Clifton, NJ). 2020;2081:177–190. doi:10.1007/978-1-4939-9940-8_12 31721125

[56] Chen Q, Tang K, Zhang X, et al. Establishment of pseudovirus infection mouse models for in vivo pharmacodynamics evaluation of filovirus entry inhibitors. Acta Pharmaceutica Sinica B. 2018;8(2):200–208. doi:10.1016/j.apsb.2017.08.003 29719780 PMC5925413

[57] Weissleder R. A clearer vision for in vivo imaging. Nat Biotechnol. 2001;19(4):316–317. doi:10.1038/86684 11283581

[58] Sadikot RT, Blackwell TS. Bioluminescence imaging. Proc Am Thorac Soc. 2005;2(6):537–540, 511–2. doi:10.1513/pats.200507-067DS 16352761 PMC2713342

[59] Signore A, Mather SJ, Piaggio G, et al. Molecular imaging of inflammation/infection: nuclear medicine and optical imaging agents and methods. Chem Rev. 2010;110(5):3112–3145. doi:10.1021/cr900351r 20415479

[60] Mire CE, Geisbert JB, Borisevich V, et al. Therapeutic treatment of Marburg and Ravn virus infection in nonhuman primates with a human monoclonal antibody. Sci Transl Med. 2017;9(384):eaai8711. doi:10.1126/scitranslmed.aai8711. 28381540 PMC5719873

[61] Yang W, Li W, Zhou W, et al. Establishment and application of a surrogate model for human ebola virus disease in BSL-2 laboratory. Virol Sin. 2024;39(3):434–446. doi:10.1016/j.virs.2024.03.010 38556051 PMC11279801

[62] Schiffman Z, Garnett L, Tran KN, et al. The inability of Marburg virus to cause disease in ferrets Is Not solely linked to the virus glycoprotein. J Infect Dis. 2023;228(Suppl 7):S594-s603. doi:10.1093/infdis/jiad206 37288605

[63] Ng M, Ndungo E, Kaczmarek ME, et al. Filovirus receptor NPC1 contributes to species-specific patterns of ebolavirus susceptibility in bats. eLife. 2015;4:e11785. doi:10.7554/eLife.11785. 26698106 PMC4709267

[64] Zlotnik I, Simpson DI. The pathology of experimental vervet monkey disease in hamsters. Br J Exp Pathol. 1969;50(4):393–399. 5806429 PMC2072114

[65] Oertelt-Prigione S. The influence of sex and gender on the immune response. Autoimmun Rev. 2012;11(6-7):A479–A485. doi:10.1016/j.autrev.2011.11.022 22155201

